# Field and Molecular Epidemiology: How Viral Sequencing Changed Transmission Inferences in the First Portuguese SARS-CoV-2 Infection Cluster

**DOI:** 10.3390/v13061116

**Published:** 2021-06-10

**Authors:** Nicole Pedro, Veronica Fernandes, Bruno Cavadas, João Tiago Guimarães, Henrique Barros, Margarida Tavares, Luisa Pereira

**Affiliations:** 1 i3S, Instituto de Investigação e Inovação em Saúde, Universidade do Porto, 4200-135 Porto, Portugal; npedro@ipatimup.pt (N.P.); vfernandes@ipatimup.pt (V.F.); bcavadas@ipatimup.pt (B.C.); 2 Ipatimup, Instituto de Patologia e Imunologia Molecular, Universidade do Porto, 4200-135 Porto, Portugal; 3 ICBAS, Instituto de Ciências Biomédicas Abel Salazar, Universidade do Porto, 4050-313 Porto, Portugal; 4 CHUSJ, Centro Hospitalar Universitário S. João, 4200-319 Porto, Portugal; jtguimar@med.up.pt (J.T.G.); margarida.tavares@chsj.min-saude.pt (M.T.); 5 FMUP, Faculdade de Medicina da Universidade do Porto, 4200-319 Porto, Portugal; henrique.barros@ispup.up.pt; 6 EPIUnit, Instituto de Saúde Pública, Universidade do Porto, 4050-091 Porto, Portugal

**Keywords:** COVID-19, infection cluster, viral sequencing, epidemiological survey, family versus work environment

## Abstract

Field epidemiology and viral sequencing provide a comprehensive characterization of transmission chains and allow a better identification of superspreading events. However, very few examples have been presented to date during the COVID-19 pandemic. We studied the first COVID-19 cluster detected in Portugal (59 individuals involved amongst extended family and work environments), following the return of four related individuals from work trips to Italy. The first patient to introduce the virus would be misidentified following the traditional field inquiry alone, as shown by the viral sequencing in isolates from 23 individuals. The results also pointed out family, and not work environment, as the primary mode of transmission.

## 1. Introduction

Epidemiology [1,2,3] and phylogeography [4,5,6] contribute to the understanding of the dynamics of infections, and the major role of superspreading events in the introduction and dispersion of SARS-CoV-2. Epidemiological data collected in the beginning of the pandemic [1] pointed to an astounding possibility of a mere proportion of 20% of infected individuals being enough to cause 80% of the new infections. The phylogeographic analyses of thousands of SARS-CoV-2 sequences across the globe also allowed us to identify multiple founder effect episodes in the COVID-19 pandemic, which were associated with superspreader hosts [4]. As the pandemic progresses, there is a continuous dynamic tracking of emerging variants on the thousands of SARS-CoV-2 sequences being regularly published [7]. Recently, the appearance of the B.1.1.7/20I/501Y.V1 lineage (first detected in the UK), the B.1.351/20H/501Y.V2 lineage (first detected in South Africa), the B.1.1.28/20J/501Y.V3/P.1 lineage (first detected in Brazil) and the B.1.617 lineage (first detected in India) is revealing new variants that seem to have a higher infectious transmission rate [6,8,9,10], contributing to further, and eventually more serious, superspreader events. Their accurate description is essential to understand the dynamics of the infection and the impact of preventive measures.

When applied to a particular transmission chain, viral sequencing has the potential to adequately confirm the transmission events, but despite recent technological advances, a joint field epidemiology and viral sequencing strategy remains poorly explored [11,12]. An example of the application of this joint approach was conducted in nursing facilities, [13,14,15] helping to clarify the role of cohabitation in the transmission and the high risk of outbreaks in this kind of institution. In addition, the study of a widespread SARS-CoV-2 infection in a correctional facility [16] showed that it is a challenge to control COVID19 outbreaks in these types of facilities, due to the difficulty in maintaining inmates’ social distance when at or close to full capacity, and in controlling movements from outside and within the institution. As such, this joint approach to study how transmission occurs in closed environments, as well as others, such as long-haul flights [17,18] and bars [19], allow for the continuous review and update of the necessary protocols to prevent infection.

Here we describe a case-study following the return of four related individuals from work trips to Italy that had the advantage of allowing to compare between the role played by work and family environments on the SARS-CoV-2 transmission.

## 2. Materials and Methods

### 2.1. Number of Individuals and RT-PCR Test

SARS-CoV-2 RNA samples were obtained from 22 out of 59 individuals linked (close family or work contacts, identified here at random as P1–P59) to this COVID-19 cluster. The RNA used for viral whole-genome-sequencing was extracted with the QIAcube extractor by using the spin-column QIAamp virus minikit (Qiagen, Hilden, Germany). RT-PCR results for SARS-CoV-2 were obtained using the LightCycler^®^ Multiplex RNA Virus Master at a LightCycler^®^ 480 Instrument II (Roche Life Science, Penzberg, Germany).

### 2.2. Viral Whole-Genome-Sequencing

For the RNA samples, PCR enrichment of the SARS-CoV-2 genome was performed using the Ion AmpliSeq^TM^ SARS-CoV-2 Research Panel, library construction with the Ion AmpliSeq^TM^ Library Kit Plus, library quantification and size range verification at the 2200 TapeStation Automated Electrophoresis System, using the High Sensitivity DNA ScreenTape (Agilent Technologies, Santa Clara, CA, USA) and next-generation sequencing on the Ion S5XL system with the Ion 530™ chip. Raw data were extracted with the Ion Torrent pipeline and the bioinformatics analysis for alignment was based on [20]. Briefly, alignment of the raw data versus the reference genome (accession number NC_045512.2) was performed with the BWA tool, and FreeBayes, BCFtools and GATK were used for variant calling. Variant annotation was made with SnpEff and the consensus sequence was inferred with BCFtools [21]. Merging of the consensus sequences with publicly available SARS-CoV-2 whole genomes from ViPR [22], and using MAFFT [23], we obtained the phylogenetic analysis and lineage/clade affiliation (first 130 bp and last 50 bp were masked). The rooted phylogenetic tree was obtained with IQ-TREE 2 [24] and visualized using Interactive Tree of Life version 4 [25].

### 2.3. Serological Tests

RT-PCR diagnosis date was considered a proxy for date of infection. Three months later, a serological test with a chemiluminescent microparticle immunoassay for the qualitative detection of IgG against the SARS-CoV-2 nucleoprotein (Abbott Diagnostics, Chicago, IL, USA) was offered to all cases and their household contacts, and 43 accepted it. Samples were run on the Abbott Architect instrument following the manufacturer’s instructions. 

## 3. Results

### 3.1. Case Study

**Figure 1:**
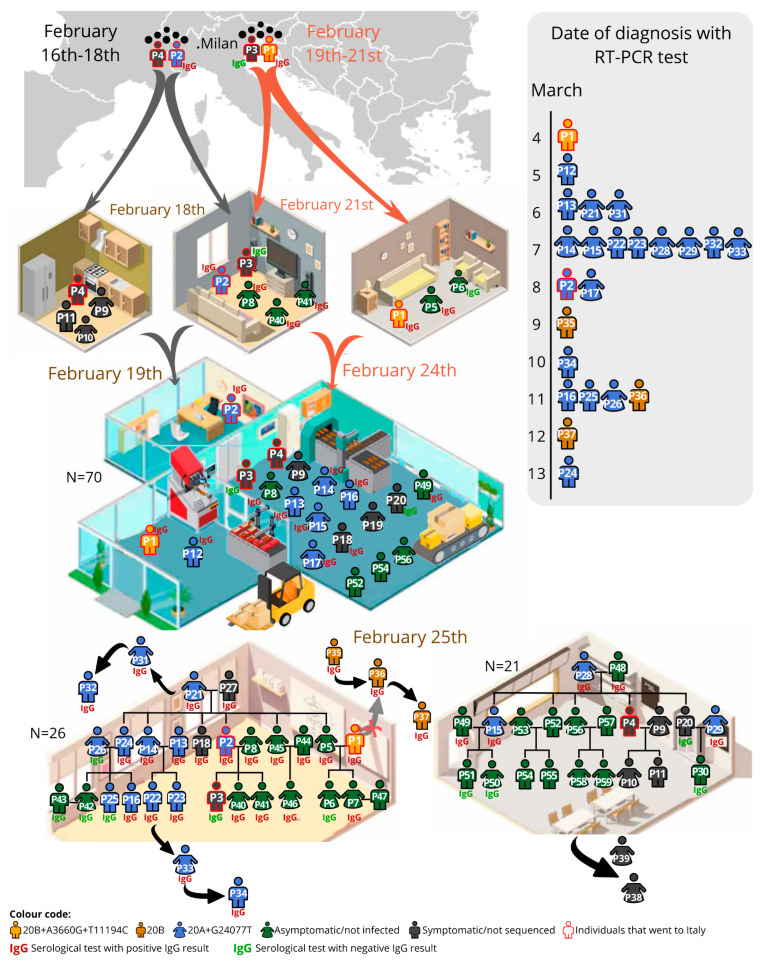
Representation of the studied SARS-CoV-2 in the 59-individual cluster in the diverse environments (at home, factory, two distinct family gatherings and external events). Date of diagnosis is represented in the lateral bar. The code of colours mean: in yellow individuals infected by the 20B+A3660G+T11194C viral haplotype; in gold the 20B haplotype; in blue the 20A+G24077T haplotype; in grey the symptomatic individuals, not sequenced; in green the not-infected/asymptomatic; the red outline indicates the individuals who went to Italy. Besides the family and factory, other close contacts were established based on the epidemiological survey: friends of P1 (P35–P36–P37); family of the girlfriend of P22 (P33–P34); family of a friend of P21 (P31–P32); other family members of P29 (P38–P39).

Four family-related individuals (P1–P4; Figure 1) travelled to Milan for work: two between February 16th–18th (P2 and P4, brothers-in-law) and two between February 19th–21st (P3, the son of P2, and P1, brother-in-law of P2). P2 owns a 70-worker factory located in a small community; 16 of those workers had a family connection with the factory owner. On their return, the four individuals had daily contact in the factory and were present in two large lunches (carnival) on February 25th. As the first reported COVID-19 cases in Portugal dated from the 1st of March 2020, no safety measures were implemented anywhere in the country by February 2020.

### 3.2. Description of the Transmission Chain

As these cases were detected in the initial phase of the Portuguese epidemic, and the recommendation from health officials at that time was to admit all COVID-19 infections, the majority of the confirmed infected individuals of this cluster had a brief inpatient stay at the reference hospitals. However, none of these cases were considered severe and none required major medical care. 

The first individual to be molecularly diagnosed, on March 4th after five days of symptoms, was P1, and thus considered the index case. However, P2, diagnosed on March 8th, was symptomatic since February 23rd; P3, though mildly symptomatic after arriving from Italy, was never tested (the serology was negative); P4 had influenza-like symptoms but did a late RT-PCR test on March 16th, which was negative (no serology available). 

Only 3 out of the 54 non-family-related co-workers developed symptoms and had a positive RT-PCR (P12–P17–P19; P12 and P17 provided a blood sample and had a reactive serological test), in comparison with 11 out of 16 family-related co-workers (additionally, the asymptomatic P8 and P49 were later shown to be IgG-reactive). The social gatherings probably contributed to most of the infection transmissions: in Family1 (*n* = 26) there were 13 infected individuals (including P1–P2) confirmed by RT-PCR, one symptomatic not tested (P3) and eight asymptomatic but IgG-reactive; in Family2 (*n* = 21) there were five RT-PCR confirmed cases, and two asymptomatic but reactive for IgG.

The serology results obtained for the individuals in our cluster are in agreement with current studies. USA household members of front-line essential workers that tested positive for SARS-CoV-2 detected IgG antibodies in 80% of cohabitant individuals, suggesting that there is a high rate of transmission among household members [26]. This explains the IgG positive results in the majority of the individuals of this cluster, even among the asymptomatic cases. In symptomatic individuals and previously confirmed cases of SARS-CoV-2 that remained seronegative, such as P3, P20, P25 and P26, the immunity may be mediated through T cells. In these cases, it may be necessary to investigate additional humoral and cellular responses to detect virus-specific T cells as proof of a SARS-CoV-2 infection [27,28].

### 3.3. Viral Sequencing Interpretation

**Figure 2:**
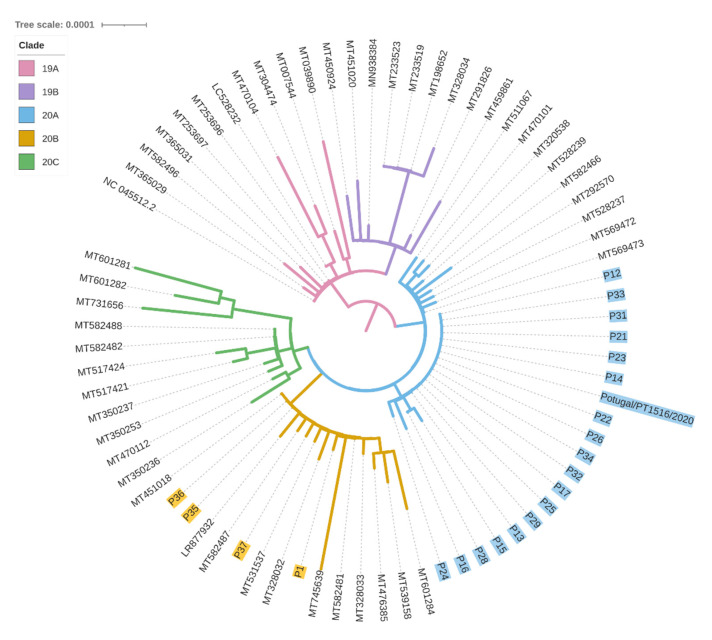
Phylogenetic tree of the SARS-CoV-2 whole sequences isolated from the samples analysed here and other sequences from GenBank.

The sequences analysed were affiliated with two major clades: 20A clade, defined by the 241T, 3037T, 14408T and 23403G mutations, and 20B clade, defined by the 241T, 3037T, 14408T, 23403G, 28881A, 28882A and 28883C mutations. P1 SARS-CoV-2 sequence was affiliated with 20B clade (basal sequence+A3660G+T11194C variants; Figure 2). None of the other 21 sequenced samples displayed such a viral sequence: 18 were affiliated with 20A clade (basal sequence+G24077T) and three with the 20B clade. These last three were friends of P1, linked to this transmission chain based on the epidemiological survey. Briefly, P35 indicated an unknown individual as the probable source of infection, however P36 indicated the contagious source as both P1 and P35, while P37 indicated P36 as the source of transmission. Through viral sequencing, it was possible to detect that all three individuals were affiliated with the 20B clade, P35 and P36 with only the basal sequence, and P37 with the additional G4432T mutation. However, the absence of the P1 additional variants in these samples implies that P35 was the most probable contagious source of this group of friends.

The viral sequence of P2 (obtained at another laboratory and identity confirmed by the providing medical doctor; available at www.insaflu.insa.pt/covid19 as Portugal/PT1516/2020, accessed on 12 November 2020) matched the 20A+G24077T dominant sequence in the transmission chain (19 individuals). Thus, P2, the owner of the factory and related to the two family clusters, was the most probable individual that initiated the superspreader event, although we cannot exclude that P3 or P4 contributed to the transmission along this chain. 

The transmission level presented in Table 1 was defined based on direct contact with the independent index cases (indicated by 0) identified in this study, P1 (no transmission), P2 (the superspreader) and P35 (in fact, an independent small chain). In this transmission chain, individuals were considered level 1 when there is evident direct contact with the index case. For that reason, all factory workers and individuals present in the Family1 lunch were considered 1st level of transmission, except for P22 and P23 who could also be 2nd level of transmission. These two were only present in the Family1 lunch (1st level of transmission), however their parents (P13/P14) as factory workers could also have been the contagious source (2nd level of transmission). As P33 and P34 are later infections from P22, their level of transmission is also variable (2nd or 3rd for P33; 3rd or 4th for P34). P28 and P29 are 2nd level transmissions and were probably infected by factory workers present in the Family2 lunch: P15 was the source for the infection of P28 (shared additional mutation), while the husband of P29 (P20) is the most likely contagious source for P29. P31 and P32 are 2nd and 3rd level of transmissions, respectively, indicated by the epidemiological surveys.

Four individuals amongst the 18 presenting the 20A+G24077T lineage had additional variants concordant with evolution along time and the level of transmission, at a proportion superior to 50% (tending to fixation; Table 1). Particularly, P15 displayed two additional mutations, T14418C and G26634T; P16 displayed two additional mutations, C2062T and C6279A; P24 presented the additional C4456T mutation, and P28 the G26634T mutation. As most of these mutations are not present in 100% frequency, they may represent intra-individual accumulation of diversity.

### 3.4. Viral Sequencing Contextualisation

**Figure 3:**
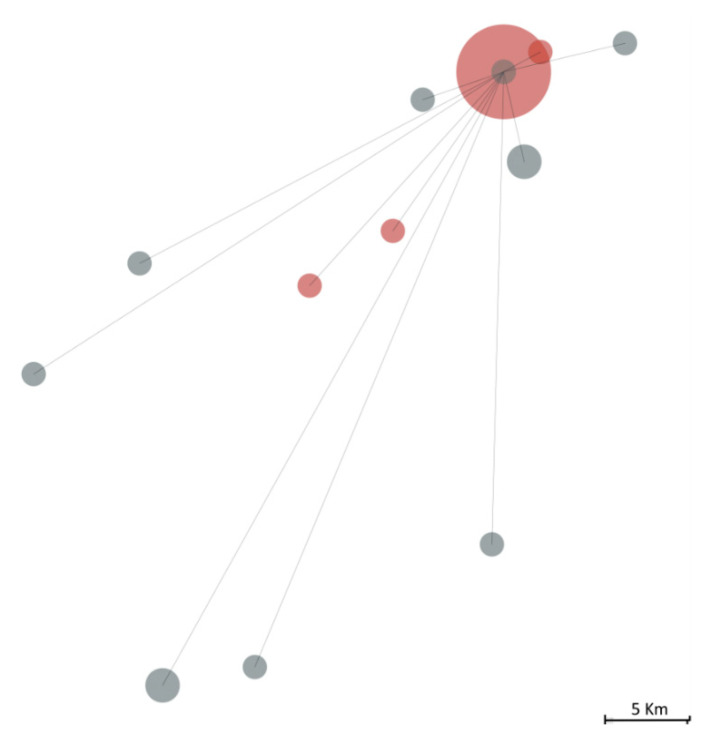
The geographic distance between individuals infected by the SARS-CoV-2 20A+G24077T lineage, including the 18 belonging to the studied infectious cluster (in red) and 11 other individuals (in grey) out of 150 Covid-19 Northern Portuguese cases identified during the same period (till 16 March 2020). The size of the circle is proportional to the effective size observed at a specific point, while lines represent the linear geographic distance from the bulk of the infectious cluster.

We further contextualised this cluster amongst other 150 Covid-19 Northern Portuguese cases identified during the same period (until the 16 March 2020) for which we performed the viral sequencing (Figure 3). The P1-20B+A3660G+T11194C lineage was not found in anyone else, while the P2-20A+G24077T lineage was observed in 11 out of those 150 cases, although no epidemiologic links were established with this cluster.

## 4. Conclusions

The viral sequencing results allowed us to clarify that the supposed index case, P1, was not in fact responsible for this superspreading situation. Family contact was the most important mode of transmission, in comparison with daily contact with non-familiar co-workers. This conclusion concurs with epidemiological observations [29], but as we have proven here, it is of paramount importance that viral sequencing is performed for proper identification of superspreading events. This viral sequencing validation is essential in order to deeply investigate the characteristics that render an infected individual responsible for a superspreading event. This would help us to better understand the pandemic and ultimately offer evidence for prevention, case finding and contact tracing. Additionally, as it occurred in this case, the misidentification of the first individual to introduce the virus led to stigma and discrimination and boosted social prejudice in the affected small community, as well as the diffusion of erroneous news nationwide by the media coverage.

## Figures and Tables

**Table 1:** Detailed information for the transmission event, level and viral affiliation and diversity in the 23 sequenced samples. * indicates the sample sequenced in http://www.insaflu.insa.pt/covid19 as Portugal/PT1516/2020 (accessed on 12 November 2020).

ID	Transmission Event			Transmission Level	Viral Sequence			
	Factory	Family1	Family2		Clade	Other Non-Clade Basal Mutations (%)		
**P1**	X	X		0	20B	A3660G (93%)	T11194C (55%)	
**P35**				0	20B			
**P36**				1 (P35)	20B			
**P37**				2 (P36)	20B	G4432T (99%)		
P2 *	X	X		0	20A	G24077T		
**P12**	X			1 (P2)	20A	G24077T (100%)		
**P13**	X	X		1 (P2)	20A	G24077T (100%)		
**P14**	X	X		1 (P2)	20A	G24077T (100%)		
**P15**	X		X	1 (P2)	20A	G24077T (100%)	T14418C (54%)	G26634T (100%)
**P16**	X	X		1 (P2)	20A	G24077T (100%)	C2062T (70%)	C6279A (65%)
**P17**	X			1 (P2)	20A	G24077T (86%)		
**P21**		X		1 (P2)	20A	G24077T (100%)		
**P22**		X		1 (P2) or 2 (P13/P14)	20A	G24077T (100%)		
**P23**		X		1 (P2) or 2 (P13/P14)	20A	G24077T (100%)		
**P24**		X		1 (P2)	20A	G24077T (100%)	C4456T (82%)	
**P25**		X		1 (P2)	20A	G24077T (98%)		
**P26**		X		1 (P2)	20A	G24077T (100%)		
**P28**			X	2 (P15)	20A	G24077T (100%)	G26634T (73%)	
**P29**			X	2 (P4/P20)	20A	G24077T (100%)		
**P31**				2 (P21)	20A	G24077T (100%)		
**P32**				3 (P31)	20A	G24077T (100%)		
**P33**				2 or 3 (P22)	20A	G24077T (100%)		
**P34**				3 or 4 (P33)	20A	G24077T (100%)		

## Data Availability

These sequences are available at GenBank (Accession Numbers: MW556267-MW556288).
